# Commentary: Human gut, breast, and oral microbiome in breast cancer: a systematic review and meta-analysis

**DOI:** 10.3389/fonc.2023.1253435

**Published:** 2023-09-19

**Authors:** Guilherme Gamba, Antonio José Grande, Tamy Colonetti, Maria Laura Rodrigues Uggioni, Leonardo Roever, Maria Inês da Rosa

**Affiliations:** ^1^Laboratory of Translational Biomedicine, Universidade do Extremo Sul Catarinense, Criciúma, SC, Brazil; ^2^Laboratory of evidence-based practice, Universidade Estadual de Mato Grosso do Sul, Campo Grande, MS, Brazil; ^3^Department of Clinical Research, Federal University of Uberlândia, Uberlândia, Brazil; ^4^Gilbert and Rose-Marie Chagoury School of Medicine, Lebanese American University, Beiruta, Lebanon

**Keywords:** microbiota, breast cancer, systematic review, meta-analysis, breast

## 1 Introduction

This text is intended as a “letter to the editor” in order to contribute to the reflection of a recent systematic review performed by Thu et al. (1). The review suggested that the fecal, tumor, and oral microbiome profiles of breast cancer patients differ in microbiota abundance by menopausal status, menarche, cancer stages, and the change in the microbial pattern before and after chemotherapy.

## 2 Commentary and discussion

However, we have observed a few inconsistencies concerning this synthesis, which we would like to point out, namely: (1) In the Introduction, there is a misconception on the definition of subtypes of breast cancer: the definition of luminal A and luminal B disease by the AJCC 8th edn; Amin et al. (2) do not include HER2-positive disease but HER2-negative disease instead. The reference they use for this concept in the systematic review is Loibl et al. (3); however, when we double-checked the original article, the concept was correct, so there was a misinterpretation of the information contained in the study of Loibl et al. (3). (2) In Table 5, the description in the study of Uzan-Ulzari et al. (4) is inaccurate because they described five patients with benign cancers when, in fact, what is described in the original study are five patients with gynecological cancer. (3) In Tables 3–5, the authors use Illumina Sequencing in the description of the microbiota detection methods, but this is a platform that performs several genomic tests, so it would be important to describe the specific test because, in the way presented, we have a false impression that all studies used the same test, which is false as Zhu (5) used a metagenomic sequencing test and most other studies used 16S rRNA sequencing. (4) The study by Goedert (6) uses standard error, and the study by Byrd (7) uses standard deviation. When using both in the forest plot, we observed that SE was not converted to SD in the analysis. According to the study of Kadlec et al. (8), using standard error instead of standard deviation can overstate values, producing large effect sizes and overly narrow confidence intervals. The standard deviation can be estimated from reported standard errors of the mean by multiplying by the square root of *n*, it may play an aggravating factor since the heterogeneity found was *I*^2 = ^87%. According to the Cochrane Handbook (9), it is not recommended to conduct a meta-analysis because heterogeneity can affect the validity and interpretability of the meta-analysis results. There are only a small number of studies available or if the studies have limited sample sizes, it may not be appropriate to conduct a meta-analysis. In such cases, it is better to rely on the results of individual studies or consider other forms of evidence synthesis.

Meta-analysis is a powerful statistical approach that combines data from multiple individual studies to examine specific research questions or hypotheses. However, it is important to be aware of potential sources of error that can impact the accuracy and reliability of the calculated results. These errors can ultimately lead to flawed conclusions if not properly addressed and accounted for in the analysis. Therefore, appropriate statistical methods are necessary to minimize the risk of statistical errors and ensure the validity of the meta-analysis findings.

## Author contributions

GG: Conceptualization, Investigation, Writing – original draft. AG: Conceptualization, Methodology, Writing – original draft. TC: Conceptualization, Methodology, Writing – original draft. MU: Conceptualization, Visualization, Writing – review & editing. LR-D: Conceptualization, Resources, Writing – review & editing. MR: Conceptualization, Project administration, Supervision, Writing – original draft.
